# Processing Nasals with and without Consecutive Context Phonemes: Evidence from Explicit Categorization and the N100

**DOI:** 10.3389/fpsyg.2013.00021

**Published:** 2013-01-29

**Authors:** Heidrun Bien, Pienie Zwitserlood

**Affiliations:** ^1^Institute for Psychology, Otto-Creutzfeldt Center for Cognitive and Behavioral Neuroscience, University of MünsterMünster, Germany

**Keywords:** assimilation, nasal, speech perception, N100, phonemes, underspecification, context

## Abstract

With neurophysiological (N100) and explicit behavioral measures (two-alternative forced-choice categorization), we investigated how the processing of nasal segments of German is affected by following context phonemes and their place of articulation. We investigated pre-lexical processing, with speech stimuli excised from naturally spoken utterances. Participants heard nasals (/*n*/, /*m*/, and place-assimilated /*n*′/), both with and without a subsequent context phoneme. Context phonemes were voiced or voiceless, and either shared or did not share their place of articulation with the nasals. The explicit forced-choice categorization of the isolated nasals showed /*n*′/ to be in-between the clear categorizations for /*n*/ and /*m*/. In early, implicit processing, /*m*/ had a significantly higher N100 amplitude than both /*n*/ and /*n*′/, with, most importantly, no difference between the latter two. When presented in context (e.g., /*nb*/, /*mt*/), explicit categorizations were affected by both the nasal and the context phoneme: a consecutive labial led to more M-categorizations, a following alveolar to more N-categorizations. The early processing of the nasal/+context stimuli in the N100 showed strong effects of context, modulated by the type of preceding nasal. Crucially, the context effects on assimilated nasals /*n*′/ were clearly different to effects on /*m*/, and indistinguishable from effects on /*n*/. The grouping of the isolated nasals in the N100 replicates previous findings, using magnetoencephalography and a different set of stimuli. Importantly, the same grouping was observed in the nasal/+context stimuli. Most models that deal with assimilation are either challenged by the mere existence of phonemic context effects, and/or use mechanisms that rely on lexical information. Our results support the existence, and early activation, of pre-lexical categories for phonemic segments. We suggest that due to experience with assimilation, specific speech-sound categories are flexible enough to accept (or even ignore) inappropriate place cues, in particular when the appropriate place information is still present in the signal.

## Introduction

Speech perception is a fast and efficient process. A continuous, highly variable, stream of sounds with no clear boundaries is perceived as words, forming utterances. The sounds occur as waves composed of frequencies within the range of hearing. Perception works remarkably well, despite the lack of invariance between input and perceived phonemes (e.g., Liberman et al., 1967). According to models of categorical perception, speech is perceived in distinct classes, with sharp boundaries between categories (e.g., Repp and Liberman, 1987; Damper and Harnad, 2000). We label these categories as phonemes, defined by the International Phonetic Association as the smallest segmental unit of sound employed to form meaningful contrasts between utterances. The realizations of a given phoneme can vary considerably due to factors such as differences between speakers, changes within speakers (emotional states, levels of fatigue, etc.), and the variance of phonemes themselves (e.g., Cutler and Clifton, 1999). In addition, the realization of a phoneme can be affected by neighboring, that is, preceding or subsequent speech segments. Due to co-articulation, phonemes can overlap and their features (such as the place of articulation, or voicing) can assimilate. In regressive assimilation of place, a phoneme adopts the place of articulation (henceforth PoA) of the following phoneme (the alveolar /*n*/ in “*rainbow*” can adopt the PoA of the following labial /*b*/, leading to the pronunciation “*raimbow*”). The occurrence of regressive assimilation of place is asymmetric in natural speech, as alveolar segments tend to assimilate to non-alveolar segments, but only rarely vice versa (e.g., Lahiri and Marslen-Wilson, 1991; Marslen-Wilson et al., 1995). Assimilation is more likely to occur when the speech rate is high (e.g., Läufer, 1995).

The consequence of variation in speech is a failure of one-to-one mapping of sound realization and phonemic identity (e.g., Cutler and Clifton, 1999). Variation in the speech signal can be accommodated by flexible lexical representations that are able to deal with deviance, a topic that has gained much interest during the last decades (e.g., Lahiri and Marslen-Wilson, 1991; Lahiri and Reetz, 2002). Another possibility is flexibility at pre-lexical levels, where speech input is represented with increasing specificity and invariance (e.g., Obleser and Eisner, 2009; DeWitt and Rauschecker, 2012). There is ample evidence for pre-lexical categorization of speech sounds (e.g., Repp, 1984; Damper and Harnad, 2000), and for distinct neural codes for such categories in auditory cortex (Eulitz and Lahiri, 2004; Bien et al., 2009; Obleser and Eisner, 2009; Scharinger et al., 2012). Our main aims are (1) to provide corroborating evidence for pre-lexical categorization of speech, (2) to assess effects of assimilation due to adjacent phonemes on sub-lexical categorization, both in (3) the presence and absence of such neighboring phonemes.

To this aim, we examined the influence of following context phonemes (/*b*/, /*d*/, /*p*/, /*t*/) on the perception of assimilated and non-assimilated nasals in German. The German phoneme inventory contains three nasal consonants (/*m*/, /*n*/, /η/). They are classified according to their PoA, which is labial for /*m*/, alveolar for /*n*/, and velar for /η/. We used tokens of /*m*/, /*n*/, and labially place-assimilated /*n*′/. Segments of natural speech were cross-spliced to create nasal/+context segments that were either congruent (/*nd*/, /*nt*/, /*mb*/, /*mp*/), incongruent (/*md*/, /*mt*/, /*nb*/, /*np*/), or unclear (/*n*′*d*/, /*n*′*t*/, /*n*′*b*/, /*n*′*p*/) with respect to their place of articulation. In natural speech, a change of PoA does not occur at random but only in appropriate phonemic context. We assessed both early, implicit measures (N100, EEG), and the explicit, forced-choice categorization of nasals, presented in isolation, or with a following context phoneme. As mentioned earlier, variation in the input is often dealt with by means of flexible lexical representations. Models of lexical representation and processing differ in how this flexibility is implemented, and – importantly – in whether phonemic context plays a role. One model that allows for a less than perfect match between input and lexical representation is the TRACE model of spoken-word recognition (McClelland and Elman, 1986). It incorporates featural, phonemic, and lexical levels with bi-directional, excitatory connections. The input *raim* activates word nodes such as *rain*, *rail*, or *same*. Recognition might be delayed, but variation in the input does not cause severe problems, unless there is a closer match with another word form. The PoA-asymmetry is not modeled in TRACE: the degree of deviance matters, and it matters for alveolars as much as it does for labials or velars. Crucially, the effect of mismatch is not assumed to be modulated by context (i.e., *raim* does not activate rain any better in *raimbow* than in *raimcoat* or *raimdrop*).

Other than TRACE, theories adhering to underspecification provide for the asymmetry in PoA-assimilation at the level of the mental lexicon. Following linguistic theories of underspecification (e.g., Kiparsky, 1985; Archangeli, 1988), redundant information can be derived by language-specific or universal default rules. Representations are abstract and not all features are specified. In PoA, alveolar is considered the default, and as such unspecified (e.g., Paradis and Prunet, 1991). The principle of underspecification was applied to lexical processing (Lahiri and Marslen-Wilson, 1991; Lahiri and Reetz, 2002). In such models, features are directly mapped onto lexical representations, which are minimally specified in their phonetic description and as such compatible with all phonological variants. FUL, the model proposed by Lahiri and Reetz (2002), assumes that alveolar segments are unspecified with respect to their PoA, while labial or velar segments are fully specified. FUL can easily explain the observed asymmetry in PoA-assimilation in natural speech. Importantly, the mapping of features to minimally specified lexical forms is context-insensitive. If a particular feature is not specified in the lexicon, a change in that feature due to assimilation is tolerated, independent of the phonemic context.

This is different in the model by Gaskell and Marslen-Wilson (1996), who also assume underspecified lexical representations. Unlike the FUL model, they propose a phonological inference process that is sensitive to the PoA of the phonemic context, and to alternative word forms activated in the lexicon, when evaluating a potential assimilation. Previous research has partly supported (e.g., Lahiri and Reetz, 2002; Wheeldon and Waksler, 2004) and partly challenged (e.g., Gaskell and Marslen-Wilson, 1996; Coenen et al., 2001; Mitterer and Blomert, 2003) the assumption of context-insensitivity in the processing of assimilated segments.

While the above solutions for non-random variation depend and rely on lexical and/or post-lexical processing, others argue for sub-lexical compensation mechanisms. Mitterer and Blomert (2003) investigated nasal/+context combinations such as “*tuimbank*” (assimilated variant of Dutch “*tuinbank*,” garden bench) with behavioral methods and EEG. They tested lexical contributions to compensation for assimilation by presenting the Dutch materials to German participants with no knowledge of Dutch. They found no evidence for lexical involvement, and argued for a pre-lexical regressive compensation mechanism. More precisely, if, for example, the signal /*m*/ is followed by another labial segment, that labial segment activates the unit corresponding to an alveolar PoA. As a result, the nasal is interpreted as /*n*/. The compensation mechanism makes it possible for “*raimbow*” to activate “*rainbow*” – to take an English example. A similar pre-lexical compensation was suggested by Gow (2003). In his feature-parsing approach, features extracted from the speech input are re-aligned to the segments they belong to. In the “*raimbow*” example, the feature “labial” of the nasal is re-aligned to the adjacent segment /*b*/. Importantly, the mechanisms suggested by Mitterer and Blomert and by Gow are both sub-lexical, and context-dependent by nature.

Given our aims and the nature of our stimuli, we now focus on the solutions that implement compensation for co-articulation at a pre-lexical level. What would be the units on which these mechanisms operate? In Gow’s (2003) feature-parsing approach, features are re-aligned to phonemic segments. Mitterer and Blomert propose a perceptual and subsymbolic locus of compensation in a model of phoneme processing that is continuous in nature. What is the evidence for these pre-lexical units? Most cognitive models of speech perception assume that sub-lexical units feed into lexical representations: features, in the FUL model (Lahiri and Reetz, 2002), features and phonemes in other models (McClelland and Elman, 1986; Norris and McQueen, 2008). Converging evidence for a hierarchy in the processing of speech comes from neurophysiological studies. Using both behavioral and magnetoencephalography (MEG) methods, Obleser et al. (2003) examined the influence of co-articulation on consonant and vowel processing by systematically varying the PoA. They found that the speech-sound mapping in the auditory cortex is highly sensitive to phonological features such as PoA and processes of co-articulation (e.g., assimilation). In our own earlier study (Bien et al., 2009), we investigated, with MEG, the implicit categorization of single assimilated nasals (alveolar /*n*/ assimilated to labial /*m*/ – but not completely so). The N100m (MEG equivalent of the N100 from EEG) showed that the assimilation information (labial PoA) present in the surface form of the nasal was ignored, and that the N100m reflected the underlying category: /*n*/ (more detail is given below). Evidence for a functional and neuro-anatomical hierarchy of cortical speech processing is summarized in the overview by Obleser and Eisner (2009), based on a compilation of peak activations from different MEG and fMRI studies. Together, there is evidence for the existence of both features and phonemic segments as pre-lexical units relevant in speech processing.

In this study, we concentrate on phonemic categories, and on the context-sensitivity of their activation, assessed by means of an early neurophysiological component: the N100. The N100 (or N1) is robust auditory component elicited about 80–120 ms after stimulus onset. With tones, it is sensitive to aspects of individual stimuli, such as stimulus onset time (stimuli with very slow onsets do not elicit an N1), stimulus intensity (decreasing stimulus intensity results in an amplitude decrease, but a latency increase of the N1), for example (see Näätänen and Picton, 1987, for an overview). By now, there is ample evidence that the N100 is sensitive to speech processing (e.g., in response to natural vowels or syllables; Obleser et al., 2003, 2004; Parviainen et al., 2005). We chose the N100 as a measure of speech processing that is both early and not susceptible to strategic considerations, which naturally accompany explicit categorization – the task used in a separate part of the experiment. The N100 is not impervious to task influences; in fact, it has been shown repeatedly that attention directed by the task affects the N100 (cf. Näätänen, 1990; Poeppel et al., 1996). In the task used here, rare vowels had to be detected, and thus differentiated from all other (nasal) stimuli (see also Obleser et al., 2003; Bien et al., 2009). This task does not differentially draw attention to any critical stimulus. To contrast this task to the explicit categorization task, we use the label “implicit” for the N100 measure.

In addition to its general sensitivity to speech contrasts, Obleser et al. (2005) attributed a more specific function to the N100, concluding that it reflects feature-mapping processes of speech sounds. Obleser et al. (2004) found that the N100m peak latency distinguished between vowel categories, primarily based on phonological features. Tavabi et al. (2007) analyzed the N100m to examine the mapping of phonological place features in consonant/+vowel perception. They suggest that the N100 reflects a transitional processing stage between auditory and abstract phonological representations. Bien et al. (2009) examined the perception of nasals (/*n*/, /*m*/, and /*n*′/ assimilated toward labial PoA), extracted from natural speech and presented in isolation. In two-alternative forced-choice tests (2AFC), the assimilated nasals /*n*′/ were predominantly categorized according to their surface form (i.e., as /*m*/). In the MEG experiment with no explicit categorization task, the same assimilated nasals elicited an N100 that was indistinguishable from that elicited by tokens of alveolar /*n*/, both of which differed from the N100 elicited by /*m*/. We suggested that any hint of an underlying alveolar is exploited in the N100, even when the segment is predominantly labial. As a consequence, the brain is not misled by changes in the surface form due to assimilation.

In sum, the N100 is a suitable tool to investigate the early, implicit categorization of incoming speech. It reflects the processing of features and their mapping onto phonemic units. It is thus particularly informative when trying to understand how the system so efficiently deals with invariance due to assimilation. Here, we use it to assess effects of context on the processing of nasals with and without a PoA-assimilation.

In the present study, we used a new and, compared to Bien et al. (2009), larger set of nasals. Again, all material was extracted from naturally spoken utterances, recorded by a male native speaker of German. We presented the nasals both with and without a following context phoneme. By means of cross-splicing, we studied the effects of both congruent and incongruent context on the processing of the nasals. Looking at both their explicit, behavioral categorization and their automatic grouping in the N100, we aimed to gain further insight in how the speech perception system deals with PoA variance due to assimilation. Our predictions were as follows. First, based on Bien et al. (2009), we expected a left-hemispheric focus for N100 effects of nasal processing. We also expected differences between explicit behavioral categorization and the more implicit N100 measure. If we replicate Bien et al. (2009), we predict a grouping of assimilated /*n*′/ with /*n*/, not with /*m*/, in the N100. Based on findings by Tavabi et al. (2009), with the mismatch negativity (MMN), we expect N100 effects of the contextual fit between nasals and consecutive phonemes.

## Materials and Methods

### Participants

Nineteen students of the University of Münster (15 female) with a mean age of 25 years (SD 5.70, range 21–42) took part in the experiment. All were native speakers of German. Seventeen were right-handed and none reported a history of neurological disorder. All participants gave written informed consent and received 15€ for their participation.

### Material analyses and pretest

We analyzed a large number of naturally produced assimilated and unassimilated nasals in terms of their acoustic (duration, pitch, formant structure) characteristics. In addition, 30 native speakers of German, from the same population as in the main experiment (mean age 21, three male), forced-choice categorized all nasals as M or N. All stimuli were extracted from recorded utterances of a male native speaker of German who was naïve with respect to the purpose of the study. Recordings were done with 44.1 kHz in a sound-proofed room using a Røde NT2-A condenser microphone and an M-Audio DMP3 preamplifier on an M-Audio Microtrack 24/96 digital recorder. The materials (Table A1 in Appendix) consisted of pseudo nouns, which were preceded by a case-marked determiner (“den,” “dem”) or preposition (“an,” “am”) and surrounded by a fixed carrier phrase (“Das war … gesagt.”; translation, keeping German word order, “That was … said.”). The pseudo nouns (e.g., “Bise,” “Tase,” “Puse”) were systematically varied with respect to the PoA of the onset (labial, alveolar), the voicing of the onset (voiced, unvoiced), and the type of vowel nucleus (/a/, /i/, /u/). All stimuli used in the main study were extracted from carrier sentences with determiner-noun sequences and /a/ as nucleus vowel, because these sentences delivered the best recordings.

Using visual inspection of Fast-Fourier Transformed spectra of the nasal closure, we located the second and third formant. A labial PoA was associated with an F3 under 2500 Hz and an F2 under 1500 Hz, whereas an alveolar PoA showed an F3 over 2500 Hz and an F2 over 1500 Hz. Crucially, assimilated nasals showed peaks in ranges that were present in both /*n*/ and /*m*/, suggesting that they contain cues of both PoAs, rather than being in-between. Additional factors distinguishing between natural /*n*/- and /*m*/-tokens were length and pitch. Labial nasals tended to be longer in duration and of lower pitch than alveolar nasals. With respect to the consecutive phoneme, all nasals tended to be higher and longer when followed by a voiced context.

### Material

Three types of nasal were tested in the main experiment: prototypical /*m*/s, prototypical /*n*/s, and assimilated /*n*′/s. To be considered as prototypical, /*n*/s and /*m*/s had to reach correct categorization rates higher than 75% (across participants) in the pretest. For a token to be classified as /*n*′/, two conditions had to be met: first, the token had to stem from an utterance in which /*n*/ was followed by a labial context (/*b*/ or /*p*/), a context in which assimilation is likely to take place. Second, mean pretest categorization had to be close to chance, that is below 60% for both /*n*/ and /*m*/ categorization. We selected a total of nine tokens per type. Given that the number of tokens classified as /*m*/ exceeded the number of tokens classified as /*n*/ and /*n*′/, we selected the /*m*/-tokens in such a way that there was a close-to-perfect match in duration (ms) and pitch (Hz) for all *nasal types* (Table 1). We also ensured that the three nasals did not differ in mean intensity. Detailed information on the individual stimuli is provided in Table A2 in Appendix. Slightly higher and shorter than the average of all /*m*/-tokens, the selected /*m*/s displayed all the prototypical characteristics in their spectra. In line with their different PoAs, the formant frequencies were higher for the /*n*/- than for /*m*/-tokens. The spectra of the /*n*′/-tokens displayed characteristics of both /*n*/ and /*m*/.

**Table 1:** **Mean duration (in ms) and pitch (Hz) of the single nasal and nasal/+context stimuli**.

	Duration (ms)	Pitch (Hz)
/*n*/	59.0 (SD 7.2)	97.3 (SD 4.7)
/*nb*/	120.4	88.8
/*nd*/	117.6	93.5
/*np*/	128.7	94.9
/*nt*/	125.5	89.2
/*m*/	62.4 (SD 9.6)	92.3 (SD 6.1)
/*mb*/	123.8	85.1
/*md*/	121.0	90.5
/*mp*/	132.1	89.3
/*mt*/	128.9	84.9
/*n*′/	64.2 (SD 8.5)	96.7 (SD 5.4)
/*n*′*b*/	125.6	88.5
/*n*′*d*/	123.5	91.1
/*n*′*p*/	133.9	92.9
/*n*′*t*/	130.7	88.4

We cross-spliced all 27 nasal tokens with four *context* phonemes. Using two selected alveolars (/*t*/ = 66.5 ms, /*d*/ = 58.6 ms) and two labials (/*b*/ = 61.4 ms, /*p*/ = 69.7 ms), we created nasal/+context stimuli that were either congruent (/*nd*/, /*nt*/, /*mb*/, /*mp*/), incongruent (/*md*/, /*mt*/, /*nb*/, /*np*/), or unclear (/*n*′*d*/, /*n*′*t*/, /*n*′*b*/, /*n*′*p*/) with respect to their PoA. On average, the nasal/+context stimuli had a duration of 126 ms (SD = 5.0). Given that identical context segments were cross-spliced onto each nasal, their mean values were the same when comparing contextual congruence (e.g., comparing /*nt*/, /*nd*/, /*mp*/, /*mb*/ to /*np*/, /*nb*/, /*mt*/, /*md*/).

In total, 135 different experimental stimuli were presented to each participant: 27 nasal tokens, each presented with four different *context* phonemes, and without any context. The experimental stimuli were complemented by 10 practice stimuli of similar structure used in warming-up trials. For the EEG N100 experiment, two /a/-tokens were extracted from the same speech material to serve as easy-to-detect targets (target 1: duration: 97.4 ms, pitch: 105.1 Hz; target 2: duration: 92.3 ms, pitch: 86.4 Hz).

### Procedure

The experiment consisted of two sessions, which were separated by 1–3 days. The first session always was the EEG N100 experiment, with a vowel-detection task (vowel /a/ interspersed between the nasals). This task, which is used to keep attention to the stimuli, involves a categorization in /a/ vs. non-/a/, but does not necessitate the categorization of individual nasals. The single nasals were presented in a separate block, preceding the nasal/+context stimuli. We blocked the stimuli because due to their shorter duration, single nasals (only one fifth of the trials) might have caused unwanted odd-ball effects. The second session was a behavioral 2AFC task on the same set of stimuli. Here, the nasals with and without context were intermixed. Both sessions were set up using the Presentation@ software package (Neurobehavioral Systems, Version 14.8).

#### EEG-session

The participants were comfortably seated in front of a monitor (Samsung SyncMaster 961, 19′′ widescreen, 75 Hz, 1280 × 1024 screen definition, 32 bit color depth). They were asked to remove potential sources of interference (mobile phone, earrings, piercings, and barrettes) and to stay in a relaxed position during the measurement. The EEG was continuously recorded with ASA (Advanced Source Analysis, version 4.7.3.1, producer ANT). Data collection and evaluation were controlled by ExMan (Experiment Manager; MS Excel worksheet with active macros). We used WaveGuard 64-channel caps with 64 electrodes and an Average Reference. Impedances were kept below 5 kΩ. The vertical electro-oculogram (EOG) was recorded from two additional electrodes placed above and below the right eye, the horizontal EOG from electrodes at the outer canthus of each eye. The EEG-signal was amplified (ExG 20×, fixed = 50 m V/V) and saved with a low-pass Butterworth filter (cutoff frequency = 0.27 × sample frequency) and no high-pass filter. The sampling rate was 256 Hz. Offline filtering was done using a 0.1–30 Hz db/oct (half-power) band-pass filter.

A white fixation cross was continuously displayed in the center of the black screen to help participants to keep fixation, to minimize eye movements. The inter-stimulus interval was jittered between 1800 and 2200 ms. To keep participants attentive to the stimuli, they were instructed to press the left mouse button whenever a target token (/a/) was presented. Overall, the EEG-session took about 1.5 h, with an experimental time of 45 min. Every 1.5–2 min there was a short break, with a variable length of 10–15 s. Every 7.5–9 min there was a longer break, lasting 1.5–2.25 min. During the breaks, we displayed a countdown of the remaining seconds enabling the participants to resume a relaxed position in time. In the first block, the 27 single nasal tokens were presented, seven times each in random order (189 trials in total). With 20 additional target trials consisting of single /a/-tokens, and regular short breaks, the first block lasted about 8 min. In the second block, the 108 nasal/+context stimuli were presented, seven times each in random order, intermixed with an additional 10% of target /a/s. With 840 trials and regular breaks, the second block lasted about 37 min.

#### Behavioral session

Participants were seated in front of a computer screen, initially displaying the instruction, and a two-button response box (RB-830 Response Pad, producer Cedrus). The stimuli were presented binaurally via headphones (Sennheiser HD 565). The forced-choice categorization task was subdivided in three blocks, and lasted about 30 min in total. In each block, the 135 different experimental stimuli (27 single nasals and 108 cross-spliced nasal/+context stimuli) were presented once and in random order. Halfway each block there was a short break of 15 s. Breaks between blocks lasted 2 min.

Every trial consisted of four elements: a blank screen for 750 ms, a fixation cross for 750 ms, the task specification, and the auditory presentation of the stimulus. The task was presented in form of a forced-choice question (Which nasal did you hear?) on top of the capital letters (e.g., N or M), labeling the button box. Preceding each trial, the blank screen followed by the fixation cross shortly interrupted the display of the task. Upon hearing the stimulus, the participants indicated their choice by button press. The nasal displayed on the left side of the screen corresponded to the left hand button, the nasal on the right corresponded to the right hand button. The assignment of N and M to the left and right side of the screen was stable within a session, but balanced across participants. The participants did not receive any feedback on their decisions. After pressing a key, the next trial was initiated. The experimental trials were preceded by 10 practice trials of similar structure.

## Results

### Behavioral data

**Figure 1:**
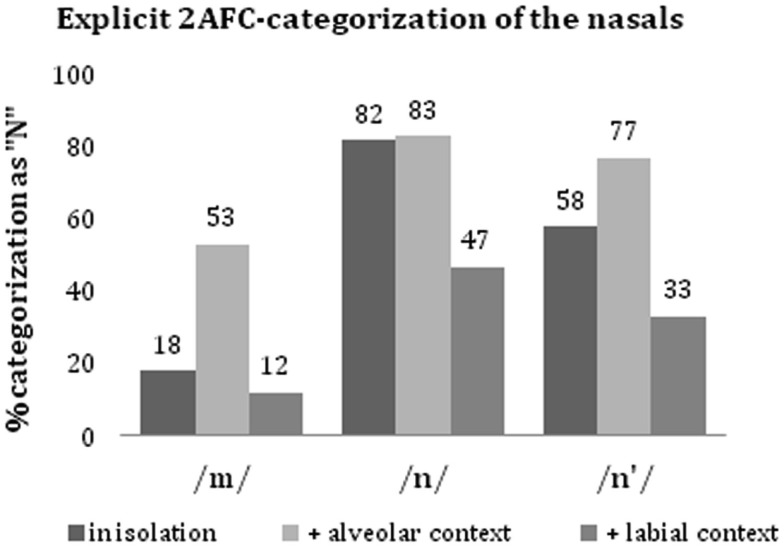
**Percentage of nasal-categorization as N in the two-alternative (N or M) forced-choice task**. The nasals (/*m*/, /*n*/, and /*n*′/) were presented in isolation, cross-spliced onto a following alveolar (/*d*/, /*t*/), or to a following labial (/*b*/, /*p*/).

**Figure 2:**
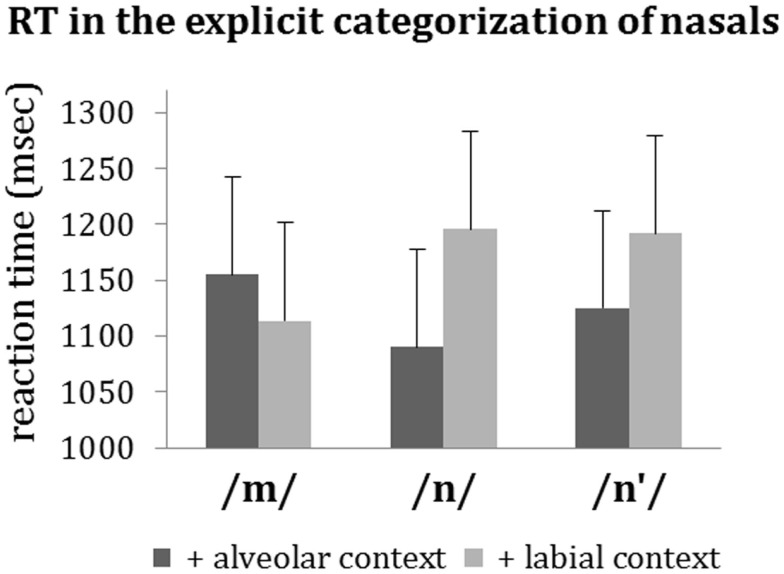
**Reaction times (and standard errors) in the two-alternative (N or M) forced-choice categorizations task**.

In the two-alternative forced-choice categorization task, the nasals had to be categorized as either N or M, summing up to 100%. Figure 1 presents the percentages of N-categorization in the crucial conditions; Figure 2 depicts the respective reaction times. We conducted analyses of variance on (a) the arcsine-transformed categorizations, and (b) the reaction times with which categorizations were made.

### Categorizations

#### Single nasals

The percentage of correct categorization was exactly 81.9% for both the /*n*/ and /*m*/-tokens, when presented in isolation. For individual participants, the percentage of correct categorization ranged from 63.0 to 96.3% (SD = 11.5%). There was no overall bias for N or M. The categorization of single /*n*′/ tokens, which cannot be labeled in terms of correctness, showed a slight preference toward N (57.9%). Note that the assimilated tokens were selected based on pretest categorization rates of maximally 60% for N and M.

#### Nasal/+context stimuli

The presentation of a following context had a strong influence on categorization, in particular when being incongruent (Figure 1). For both /*n*/ and /*m*/, the rate of correct categorization increased in congruent context (/*n*/ = 82.8%, /*m*/ = 88.0%), and declined to chance level in incongruent context (/*n*/ = 46.5%, /*m*/ = 53.1%). The categorization of /*n*′/ also changed according to the context phoneme. Followed by an alveolar context (/*d*/ or /*t*/), /*n*′/-tokens (which had been categorized as 57.9% N when presented in isolation) were predominantly categorized as N (77.1%); while the same /*n*′/-tokens were predominantly categorized as M (only 32.8% N) when followed by a labial context phoneme (/*b*/ or /*p*/). Whereas the clear nasals profited only little from congruent context, the impact of an incongruent context on their categorization was substantial. For /*n*′/-tokens, alveolar context led to a 32.7% increase in N-categorization, labial context led to a 43.4% decrease (Figure 1). An Analysis of Variance (ANOVA) on the arcsine-transformed categorization rates with the factors *Nasal* (/*m*/, /*n*/, /*n*′/), *Context-Poa* (alveolar, labial), and *Context-Voice* (voiced, unvoiced) revealed a significant main effect of *Nasal* [*F*_(2,36)_ = 30.46, *p* < 0.001, η*p*^2^ = 0.63]: /*n*/-tokens yielded a significantly higher number of N-categorizations than /*n*′/-tokens, both of which yielded a significantly higher number of N-categorizations than /*m*/-tokens. The explicit nasal-categorizations were also affected by the PoA of the context phonemes [main effect of *Context-Poa* (*F*_(1,18)_ = 24.33, *p* < 0.001, η*p*^2^ = 0.58)]: all three types of nasal were more often categorized as N when followed by an alveolar than when followed by a labial phoneme. Finally, there was a main effect of *Context-Voice* [*F*_(1,18)_ = 8.64, *p* = 0.009, η*p*^2^ = 0.32], modulated by *Context-Poa* [interaction: *F*_(1,18)_ = 19.33, *p* < 0.001, η*p*^2^ = 0.52]: nasals were more frequently categorized as N when followed by unvoiced context than when followed by voiced context (main effect *Context-Voice*), but this was true in labial context only [effect of *Context-Voice* in labial context: *t*_(18)_ = 6.23, *p* < 0.001; effect of *Context-Voice* in alveolar context: *t*_(18)_ = −0.088, *p* = 0.931].

In sum, the forced-choice categorization of the nasals was affected by both the nasals themselves and their following context phoneme. Overall, a consecutive alveolar increased the likelihood of N-categorization, while a labial context increased the likelihood of M-categorization, with weaker effects for /*p*/ than for /*b*/. Effects were strongest when the context was incongruent, and when the consecutive phonemes were voiced. Correct categorization remained high in supporting context (above 80%), but dropped by 35% in incongruent context.

##### Reaction times

There were no significant main effects of *Nasal* [/*m*/: 1121, /*n*/: 1137 ms, /*n*′/: 1157 ms; *F*_(2,36)_ = 2.365, *p* = 0.265], *Choice* [N: 1141 ms, M: 1136 ms; *F*_(1,18)_ < 1], or *Complexity* [single nasal stimuli: 1113 ms, nasal/+context stimuli: 1145 ms; *F*_(1,18)_ < 1]. For those nasals, for which categorization can be judged in terms of correctness (i.e., /*n*/ and /*m*/), correct categorizations (70.4%) were made faster (1094 ms) than incorrect ones [1250 ms, *F*_(1,18)_ = 11.425, *p* = 0.003 **]. (For the number of correct categorizations per condition, see Figure 1.)

An ANOVA on the reaction times to the nasal/+context stimuli only, with the factors *Nasal* (/*m*/, /*n*/, /*n*′/), *Context-PoA* (alveolar, labial), and *Context-Voice* (voiced, unvoiced) revealed a main effect of *Context-PoA* [*F*_(1,18)_ = 5.00, *p* = 0.038*, η*p*^2^ = 0.22] with faster reaction times for alveolar context. The effect of *Context-PoA* interacted with *Nasal* [*F*_(2,36)_ = 8.25, *p* = 0.001**, η*p*^2^ = 0.31]. Thus, context had different effects on the different types of nasal. Reaction times to /*n*/ and /*n*′/ were shorter when these nasals were accompanied by an alveolar compared to a labial context phoneme. The opposite was true for /*m*/ (Figure 2).

### EEG-data

The data were digitally filtered offline (half-power band-pass filter, 0.01–30 Hz, 24 db). Trials with activity above +75 μV or below −75 μV were rejected as artifacts. Of the 1134 events per condition, an average of 89% was accepted (for the single nasals: 1013 /*n*/, 994 /*m*/, 1012 /*n*′/). Per subject, condition, and electrode, we averaged over a time range from −0.2 to +0.6 s around stimulus onset and conducted a baseline-correction.

#### Single nasals

**Figure 3:**
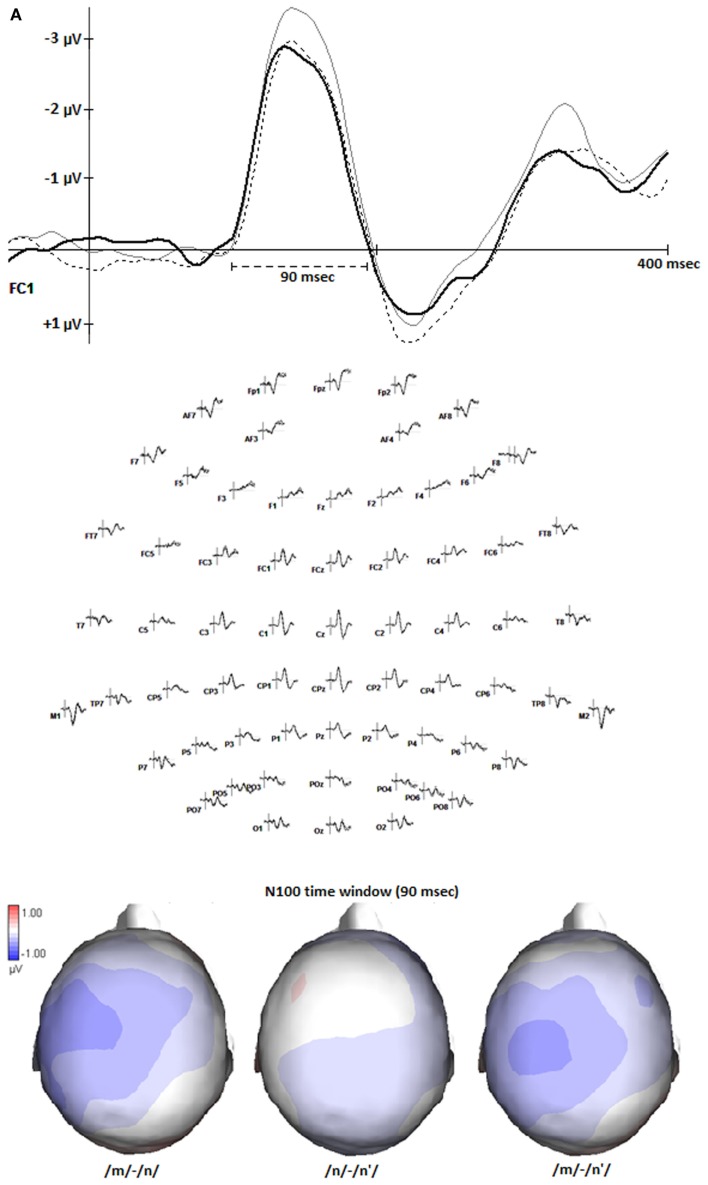

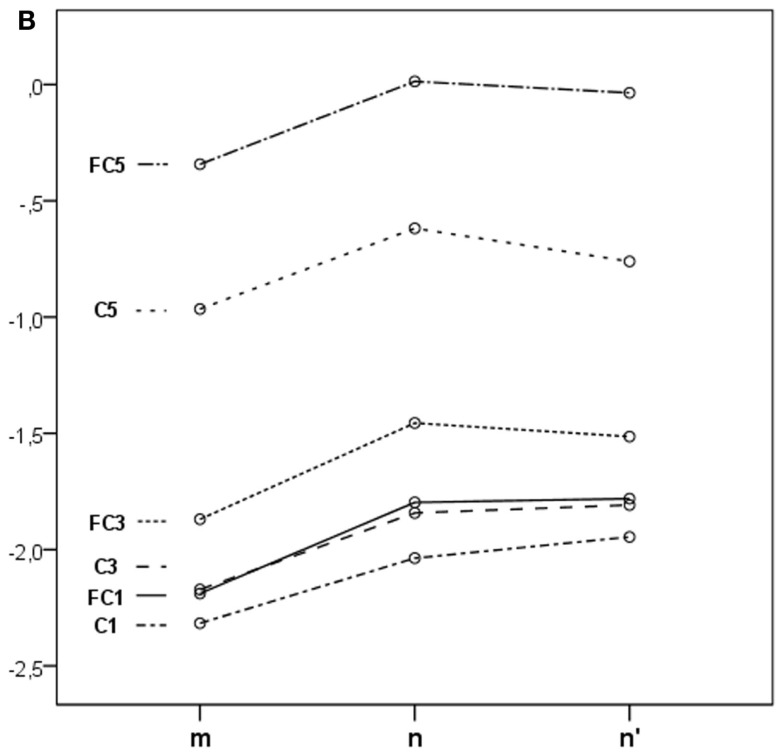
**(A)** The N100s (in μV) elicited by the nasals /*m*/, /*n*/, and /*n*′/ when presented in isolation. The ERP-graph (upper picture) shows the activation at exemplar electrode FC1, marking the N100-time window of 90 ms used for the analyses (top). All electrodes are shown in the middle. The topography (bottom) depicts the activation differences between the nasals (/*m*/-/*m*/, /*n*/-/*n*′/, and /*m*/-/*n*′/), averaged over the N100-time window. **(B)** The mean amplitudes (in μV, averaged over the N100-time window) elicited by the isolated nasals at each electrode in the selected region (FC1, FC3, FC5, C1, C3, C5).

We performed an ANOVA on the N100 mean amplitudes (a fixed time window of 90 ms around the peak) at left fronto-temporal electrodes, where the single nasals showed the greatest differences (data-driven region). Figure 3A (middle) displays the activation at all electrodes, and at exemplar electrode FC1 (top), and the mean amplitudes in the 90 ms time range in the selected region (bottom). The ANOVA with the factors *Nasal* (/*m*/, /*n*/, /*n*′/) and *Electrode* (FC1, FC3, FC5, C1, C3, C5) revealed a main effect of *Nasal* [*F*_(2,34)_ = 4.437, *p* = 0.019*, η*p*^2^ = 0.21]. The N100 mean amplitude of was higher for /*m*/ (−1.64 μV) than for both /*n*/ [−1.29 μV, *post* *hoc*: *F*_(2,34)_ = 6.761, *p* = 0.019*, η*p*^2^ = 0.29], and /*n*′/ [−1.31 μV, *post hoc*: *F*_(2,34)_ = 5.285, *p* = 0.034*, η*p*^2^ = 0.24], with no difference between the latter two [*post hoc*: *F*_(2,34)_ = 0.024, *p* = 0.880, η*p*^2^ < 0.01]. There was no interaction between *Nasal* and *Electrode* [*F*_(10,170)_ = 0.256, *p* < 0.619, η*p*^2^ = 0.02]. Figure 3B shows the mean elicited by the isolated nasals at each of the selected electrodes.

#### Nasal/+context

**Figure 4:**
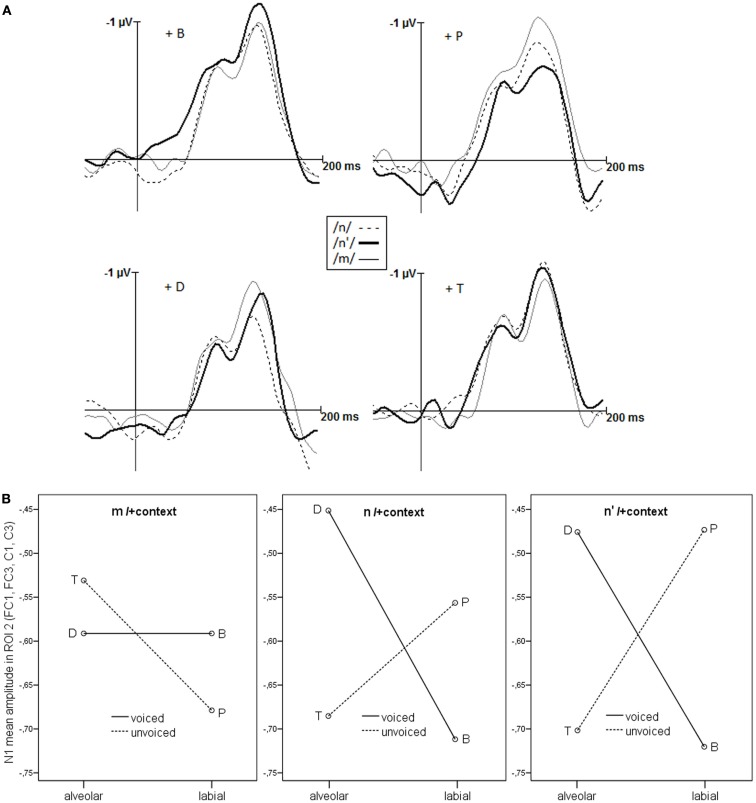
**(A)** The N100s (in μV) elicited by the nasals in context, as a function of the context phoneme (labeled P, T, B, D). The width of the N100 window is 110 ms. Only −40 ms of a −200 ms baseline are shown here. **(B)** The N100 (mean amplitude in μV) elicited by nasal/+context stimuli. Context effects are modulated by the preceding nasal.

Within the 110 ms window, the N100s elicited by the nasal/+context stimuli (Figure 4A) were slightly broader than those elicited by the single nasals, which had a width of 90 ms. Note that we cannot directly compare the amplitude of the single nasals and the nasal/+context stimuli. Single nasals were shorter in duration than nasals/+context, and presented in a separate block, ahead of the nasal/+context stimuli. With four times the number of stimuli, the second block lasted longer and the mean amplitudes were lower. As a consequence, we cannot interpret the change in amplitude from single nasal to nasal/+context stimuli, but only compare the nasal/+context stimuli to one another.

As with the single nasals, we selected the same fixed time window (110 ms) for all nasal/+context stimuli, based on the grand average. The N1 mean amplitudes, measured in μV, at the same left fronto-temporal region as for the single nasals, were then entered in an ANOVA, with the factors *Nasal* (/*m*/, /*n*/, /*n*′/), *Context-PoA* (alveolar, labial), *Voice* (voiced, unvoiced), and *Electrode* (FC1, FC3, FC5, C1, C3, C5). All main effects had *F-*values below 1, besides *Electrode* (*p* < 0.001, η*p*^2^ = 0.43). The N100 amplitudes were generally smaller at more temporal electrodes. *Context-PoA* and *Context-Voice* interacted [*F*_(1,17)_ = 10.622, *p* = 0.005**, η*p*^2^ = 0.39]. *Post hoc* tests revealed that in voiced context (/*b*/, /*d*/), the N1 amplitude of the nasal/+context stimuli tended to be higher for labial than for alveolar context [*Context-PoA* in voiced: *F*_(1,17)_ = 3.911, *p* = 0.064, η*p*^2^ = 0.19; *Context-PoA* in unvoiced: *F*_(1,17)_ = 1.432, *p* = 0.248, η*p*^2^ = 0.08]. A three way interaction of *Context-PoA*, *Context-Voice*, and *Nasal* [*F*_(2,34)_ = 3.040, *p* = 0.061, η*p*^2^ = 0.15) and a four way interaction with *Electrode* [*F*_(10,170)_ = 3.375, *p* = 0.084, η*p*^2^ = 0.17) just failed to reach significance. This interaction suggests that there may be another region that the region selected *a priori* on the basis of the nasals without context, which better reflects the differential effects of how the PoA of the context phoneme and preceding nasal interact.

An ANOVA on the N100 mean amplitudes measured in the same 110 ms time window in a smaller region (FC1, FC3, C1, C3, i.e., leaving out the most temporal electrodes), with the factors *Nasal* (/*m*/, /*n*/, /*n*′/), *Context-PoA* (alveolar, labial), *Context-Voice* (voiced, unvoiced), and *Electrode*, again, revealed no main effects (all *F*s < 1, *Electrode* *p* = 0.061, η*p*^2^ = 0.19). *Context-PoA* and *Context-Voice* interacted [*F*_(1,17)_ = 14.275, *p* = 0.002**, η*p*^2^ = 0.46]. As in the larger region, only in voiced context (/*b*/, /*d*/), the N100 amplitude elicited by the nasal/+context stimuli was higher for labial than for alveolar context [*post hoc*: *Context-PoA* in voiced: *F*_(1,17)_ = 5.221, *p* = 0.035*, η*p*^2^ = 0.26; *Context-PoA* in unvoiced: *F*_(1,17)_ = 1.114, *p* = 0.306, η*p*^2^ = 0.06]. In the smaller region, the three way interaction of *Context-PoA*, *Context-Voice*, and *Nasal* was significant [*F*_(2,34)_ = 4.427, *p* = 0.020*, η*p*^2^ = 0.21], the four way interaction with *Electrode* [*F*_(6,102)_ < 1, η*p*^2^ = 0.05] was not.

As displayed in Figure 4B, context tended to affect /*m*/ and /*n*/ differently [*post hoc*: *Context-PoA* by *Context-Voice* by /*m*/, /*n*/: *F*_(1,17)_ = 4.11, *p* = 0.059, η*p*^2^ = 0.20]. Crucially, the effects on assimilated nasals /*n*′/ were very different from effects on /*m*/ [*post hoc*: *Context-PoA* by *Context-Voice* by /*m*/, /*n*′/: *F*_(1,17)_ = 8.86, *p* = 0.008**, η*p*^2^ = 0.34], and indistinguishable from effects on /*n*/ [*post hoc*: *Context-PoA* by *Context-Voice* by /*n*/, /*n*′/: *F*_(1,17)_ < 1, *p* = 0.672, η*p*^2^ = 0.01]. For the N100 mean amplitude, elicited by /*m*/+context stimuli, context effects were non-significant [*F* < 1 for *Context-PoA* and for *Context-Voice*, *F*_(1,17)_ = 1.15, *p* = 0.298,η*p*^2^ = 0.06 for the interaction]. For both /*n*/ and /*n*′/, there was a significant, and very similar, interaction of *Context-PoA* by *Context-Voice* [*F*_(1,17)_ = 15.65, *p* = 0.001, η*p*^2^ = 0.48]. Here, the PoA of voiced and unvoiced contexts had opposite effects [*post hoc* *Context-PoA* in voiced: *F*_(1,17)_ = 7.12, *p* = 0.016*, η*p*^2^ = 0.30; *post hoc* *Context-PoA* in unvoiced: *F*_(1,17)_ = 4.55, *p* = 0.048*, η*p*^2^ = 0.21].

## Discussion

Using cross-spliced segments of natural speech, we studied the role of consecutive context phonemes on automatic and on explicit processing of non-assimilated (/*n*/ and /*m*/) and labially assimilated (/*n*′/) nasals in German. The same set of stimuli was presented in an EEG N100 experiment, and, in a later session, categorized explicitly in a 2AFC task without feedback. A selection of 27 nasal tokens, closely matched on acoustic propertied such as duration, intensity, and pitch, was cross-spliced to precede identical tokes of labial and alveolar stop consonants. The resulting set of nasal/+context stimuli included congruent (e.g., /*nd*/) and incongruent (e.g., /*nb*/) combinations as well as combinations with some conflicting information with respect to the PoA (e.g., /*n*′*d*/, /*n*′*b*/). Using both single nasals and nasal/+context stimuli, we were able to systematically analyze the influence of adjacent context phonemes on the processing of the different types of nasals. Studying both neurophysiological and behavioral data, we gained insight into the explicit categorization of the speech segments, as well as their implicit, and more automatic processing.

When presented in isolation, tokens of unassimilated nasals /*n*/ and /*m*/ yielded correct categorization rates of over 80% in the explicit forced-choice task. In line with the pretest results, the selected assimilated nasals (/*n*′/) were categorized around chance level. In implicit processing, /*n*/ and /*m*/ elicited reliably different N100 components, and crucially, the assimilated nasals grouped with /*n*/. These results, with a different and larger set of stimuli, replicate previous findings by Bien et al. (2009). There, the amplitudes elicited by /*m*/-tokens were reliably smaller than those elicited by /*n*/ and /*n*′/ tokens. In the present study, the N100 amplitudes for /*m*/-tokens were significantly larger, which is most likely attributable to the different speakers. Most importantly, in both studies the N100 reflected a reliable difference between /*n*/ and /*m*/, and the assimilated nasals grouped with the non-assimilated, alveolar nasals /*n*/. We believe that this is an important result, because it constitutes the first replication, with different materials and participants.

When presented with a following context phoneme, both the behavioral and neurophysiological data showed effects of this context. In the explicit categorization, alveolar context increased the number of N-categorizations, while labial context increased the number of M-categorizations. Incongruent context led to categorization rates near chance level, demonstrating that the adjacent context did affect, but not overrule, the effect of the nasal itself. These effects of context on the categorization of unambiguous nasals (/*n*/, /*m*/) replicates results by Mitterer and Blomert (2003), who observed a decrease of about 30% in the correctness of nasal-categorization.

The presence of a context phoneme also affected the N100 amplitudes, crucially in interaction with the type of nasal. Context had less effect on /*m*/ than on /*n*/ and /*n*′/. Importantly, again, the processing of /*n*′/+context stimuli was indistinguishable from that of /*n*/+context stimuli, and different from that of /*m*/+context stimuli.

Note that we had selected those assimilated nasals that contained characteristics of both alveolar and labial PoA in their spectra and were categorized accordingly, with no clear bias for N or M in the pretest. Assimilation is a gradual process, and many assimilated tokens will yield higher or lower percentages of M-categorizations. We selected the intermediate ones in order to maximize the conflicting information and the scope for context effects. Close to ceiling, /*n*/ and /*m*/ could not benefit much from congruent PoA information, but incongruent information reduced the categorization to chance level. Falling in-between the /*n*/- and /*m*/-tokens when presented in isolation, the categorization of the assimilated nasals showed strong effects of the context-PoA in both directions. In sum, the behavioral data reflect the pattern of PoA information delivered by both the nasals and the context phonemes. They show that, when forced to make a decision, participants base their choice on the PoA information the signal provides.

In the implicit N100 processing of the same stimuli, however, there was no gradual reflection of PoA information, but a clear grouping, in which the assimilated /*n*′/-tokens were processed as the non-assimilated /*n*/-tokens, and both clearly different from the /*m*/-tokens. The grouping was evident for both the single nasals and the nasal/+context stimuli. The task in the N100 experiment did not involve any explicit categorization of the nasals; they merely had to be distinguished from the vowel (/a/) target. In the absence of explicit categorization, the early, automatic N100 component can be assumed to reflect the normal and fast processing of incoming speech signals. The reliable grouping of assimilated nasals with alveolar nasals might reflect experience in the processing of mixed PoA information due to co-articulation. In the light of the asymmetric occurrence of PoA-assimilation, it is very efficient to exploit any alveolar information and to implicitly categorize the segment accordingly.

In the present study, we also observed effects of context on the N100 measure. Given that the primary auditory systems are located in Brodmann area 41 in the superior temporal lobe, it is not surprising that we found stronger effects by excluding the more inferior electrodes. The location of the data-driven region (see Figure 3A) fits with the assumption that speech processing is more prominent in the left hemisphere, even when assessed with the N100 (Parviainen et al., 2005). Context affected the nasals differently, with no reliable impact on /*m*/. For /*n*/ and /*n*′/, there were strong and very similar context effects, that changed direction as a function of the voicing of context segments. Whereas a voiced congruent context (nd) reduced the amplitude of the N100, a voiceless congruent context (nt) increased its amplitude. The pattern for the incongruent contexts was exactly reversed. We have no easy explanation for this pattern, but like to offer some information and speculations. First, it is unclear what a positive effect of the congruence between nasal and context phoneme would look like. One might expect the N100 to be more pronounced for congruent cases – this is what the voiceless contexts show. Alternatively, the N100 for the congruent might be more reduced. The MMN, a somewhat later component of EEG or MEG, indeed shows reductions in congruent as compared to incongruent contexts (Mitterer and Blomert, 2003; Tavabi et al., 2009). If reduction were the expected pattern, the voiced contexts show the desired effect. Unfortunately, we have no way of knowing what the appropriate pattern might be. Except for a study by Flagg et al. (2006), who found an N100-latency effect for (in)congruency of nasals and nasalized or oral vowels, we know of no study that investigated such issues with N100 amplitude. Whatever the appropriate pattern, we obtained an interaction with Context-Voice. Our materials were constructed such that N100 effects of individual nasals, and of the contextual congruence between nasals and context phonemes were independent of acoustic aspects of the stimuli. The interaction with voice, however, could be due to acoustic differences, which fits with the observation voiced and voiceless plosives elicit different N100 patterns (Steinschneider et al., 2005). Possibly, because of the brief devoicing before the burst, the unvoiced phonemes (/*p*/ and /*t*/) were sufficiently distinguishable from the preceding nasals, enabling a compensation mechanism (e.g., Mitterer and Blomert, 2003) to work. There was no compensation effect in the explicit categorizations of the nasal/+context stimuli, but the PoA by Voice interaction, with weaker context effects for /*p*/ compared to /*b*/, might point in the same direction. However, Mitterer et al. predict the same compensation mechanism for incoming labials, to such a degree that even a true /*m*/ can be mistaken as alveolar. On the contrary, we find that unvoiced context affects the N100 in the same, incongruent way when following /*n*/, but not at all when following /*m*/. Note however, that even though the N100 elicited by nasal/+context stimuli is modulated by features of the context phoneme, a compensation mechanism or phonological inference might be reflected in later processing stages.

How do the results fit with models and mechanisms proposed for dealing with variation in speech? Psycholinguistic theories adhering to underspecification (e.g., Lahiri and Reetz, 2002) predict /*n*′/ to be processed as /*n*/, and different from /*m*/. Alveolar, the default PoA, is not specified. Therefore, /*n*/ can easily adopt the PoA of the following context phoneme, without hindering lexical access. Labials, in contrast, are specified as such and may not change their PoA. This seems to fit well with our data for nasals presented in isolation, but the mere existence of context effects, as observed here, challenges underspecification views. In general, given that we used nasal/+context stimuli excised from multiword utterances, there is little solace for lexical solutions to the problem of variance – in our case, of place of articulation assimilation. Other proposals such as the compensation process proposed by Mitterer and Blomert, or the feature-parsing approach put forward by Gow (2003) are much more suitable to explain sub-lexical effects of context on the perception of phonemic segments. Note however, that the consistent grouping of the assimilated nasals, /*n*′/, with /*n*/, and not with /*m*/ in the early and automatic N100 measure occurs with and without context. The compensation view by Mitterer and Blomert is not concerned with segments in isolation, but the feature-parsing view could explain our results. Given that there is alveolar and labial information in the assimilated nasals, feature-parsing would detect both. What we wish to argue – as we did before (Bien et al., 2009) – is that any alveolar information leads to the categorization as an alveolar segment. But there is an asymmetry: any labial information does not lead to a categorization as a labial segment. What this implies is that the category “alveolar nasal” accepts more “noise” (some labial PoA, for example) whereas the category “labial nasal” is more narrow and restricted with respect to what counts as positive evidence.

The present study has a few limitations which should be noted. Using tokens extracted from natural speech both has advantages and disadvantages. By presenting real speech segments, we aimed for a better understanding of the challenges listeners face, and of their efficient processing. At the same time, we must accept less control of our stimuli. Compared to synthesized ones, natural segments will always contain information and variation that we can, at best, describe and use for selection. To reduce the variation in less relevant characteristics, the stimuli for the main study were selected such that they were closely matched in length, intensity, and pitch. The majority of our participants were psychology students in their early 1920s, as it is the case with most experimental studies run at universities. In the processing of pre-lexical speech stimuli, we believe that this group is sufficiently representative of a broader sample, particularly in the automatic processing in the N100.

In sum, we obtained clear evidence for the early categorization of assimilated nasal segments as alveolar nasal /*n*/. Despite the fact that they contain labial information, and were classified between N and Min the pretest, the assimilated nasals went along with the alveolars in all measures based on the N100 component of the EEG. The consistent grouping of /*n*′/ with /*n*/ in automatic processing fits well with theories that implements underspecification. But we also observed clear context effects of the place (and manner) of articulation of adjacent segments, which are difficult for some underspecification-based models. The exact influences of context, reflected in the N100, which were most prominent for /*n*/ and /*n*′/, cannot be easily related to phonological inference or the assumption of a regressive compensation mechanism. Again, and most importantly, the assimilated nasals behaved as alveolar *n*-segments in all early and automatic measures.

## Conflict of Interest Statement

The authors declare that the research was conducted in the absence of any commercial or financial relationships that could be construed as a potential conflict of interest.

## Appendix

**Table A1:** **The sentences used for recording**.

Das war an/am/den*/dem* Base*/Bise/Buse gesagt
Das war an/am/den*/dem* Dase*/Dise/Duse gesagt
Das war an/am/den*/dem* Pase*/Pise/Puse gesagt
Das war an/am/den*/dem* Tase*/Tise/Tuse gesagt

*All stimuli used in the main study were extracted from sentences marked with **.

English gloss: *“That was next to*/*to the … said.”*

**Table A2:** **Duration and intensity of the selected 27 tokens of prototypical /*n*/, /*m*/, and /*n*′/**.

Single nasal tokens						
	Duration (ms)			Intensity (dB)		
	/*n*/	/*m*/	/*n*′/	/*n*/	/*m*/	/*n*′/
	52	70	65	87.7	87.3	87.1
	59	76	59	86.2	88.6	87.7
	71	72	69	88.1	87.4	87.2
	60	58	44	86.9	86.8	86.6
	56	46	72	87.7	87.3	87.0
	62	57	64	87.6	88.2	88.4
	63	56	69	87.8	87.7	87.7
	63	67	69	87.6	88.1	87.0
	46	58	68	88.2	86.8	87.4
Mean	59.11	62.22	64.33	87.53	87.58	87.34
SD	7.18	9.57	8.51	0.64	0.64	0.55
